# Supplementary material for: An immune checkpoint score system for prognostic evaluation and adjuvant chemotherapy selection in gastric cancer
Source: Nat Commun. 2020 Dec 11;11:6352. doi: 10.1038/s41467-020-20260-7 (PMC7732987; doi:10.1038/s41467-020-20260-7)
Supplement: Supplementary file 1 — Supplementary Information [file 41467_2020_20260_MOESM1_ESM.pdf]

Supplementary Figure 1

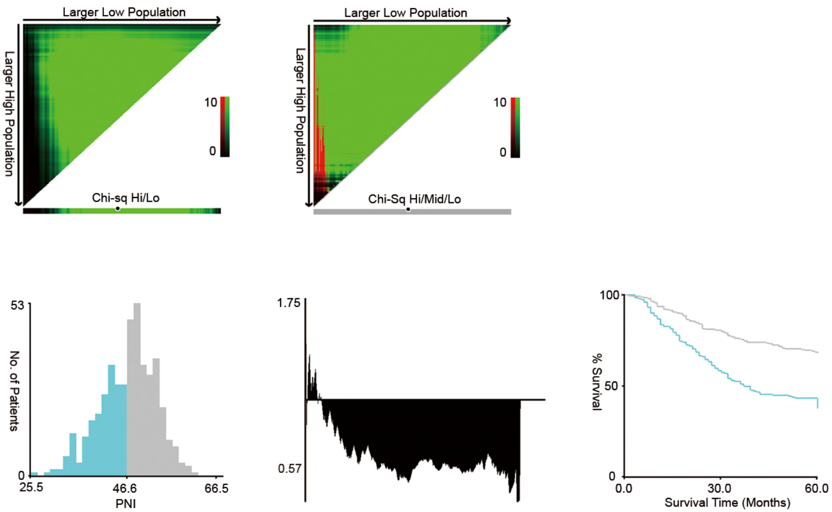

Subpopulation Cutpoints:

Pt No	% Tota	Events	Rate	Rank	Range
191	43.02	108	56.54	0 to 138	25.45 thru 46.60
253	56.98	80	31.62	139 to 288	46.65 thru 66.52
444	100.00	188	42.34	0 to 288	25.45 thru 66.52

Statistics:

Variable	Value	
Miller-Seigmund P	<0.0001	Max: <0.0001
Chi-sq Hi/Lo	33.2834	Max: 33.2834
Relative Risk 1 vs 2	1.79 / 1.00	

Supplementary Figure 1 X-tile for the PNI. The process of setting the PNI cut-off point using X-tile.

Supplementary Figure 2

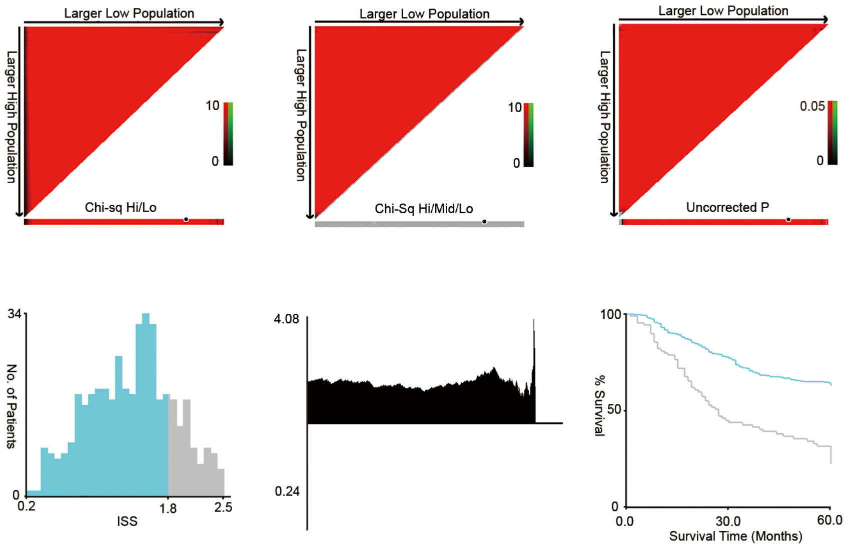

Subpopulation Cutpoints:

Pt No	% Tota	Events	Rate	Rank	Range
355	79.95	126	35.49	0 to 333	0.18 thru 1.83
89	20.05	62	69.66	334 to 410	1.83 thru 2.48
444	100.00	188	42.34	0 to 410	0.18 thru 2.48

Statistics:

Variable	Value	
Uncorrected P	<0.0001	Max: <0.0001
Miller-Seigmund P	<0.0001	Max: <0.0001
Chi-sq Hi/Lo	45.9098	Max: 45.9098
Relative Risk 1 vs 2	1.00 / 1.96	

Supplementary Figure 2 X-tile for ISS_{GC}. The process of setting the ISS_{GC} cut-off point using X-tile.

Supplementary Figure 3

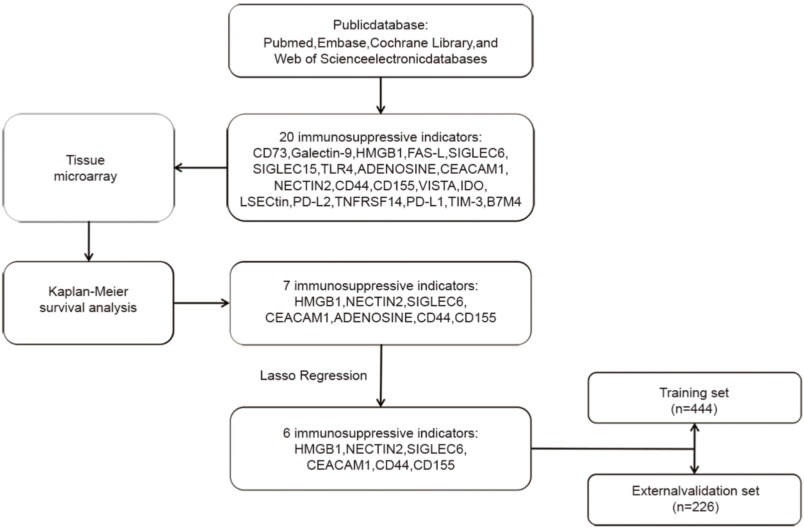

Supplementary Figure 3 Flow chart of this article.

Supplementary Figure 4

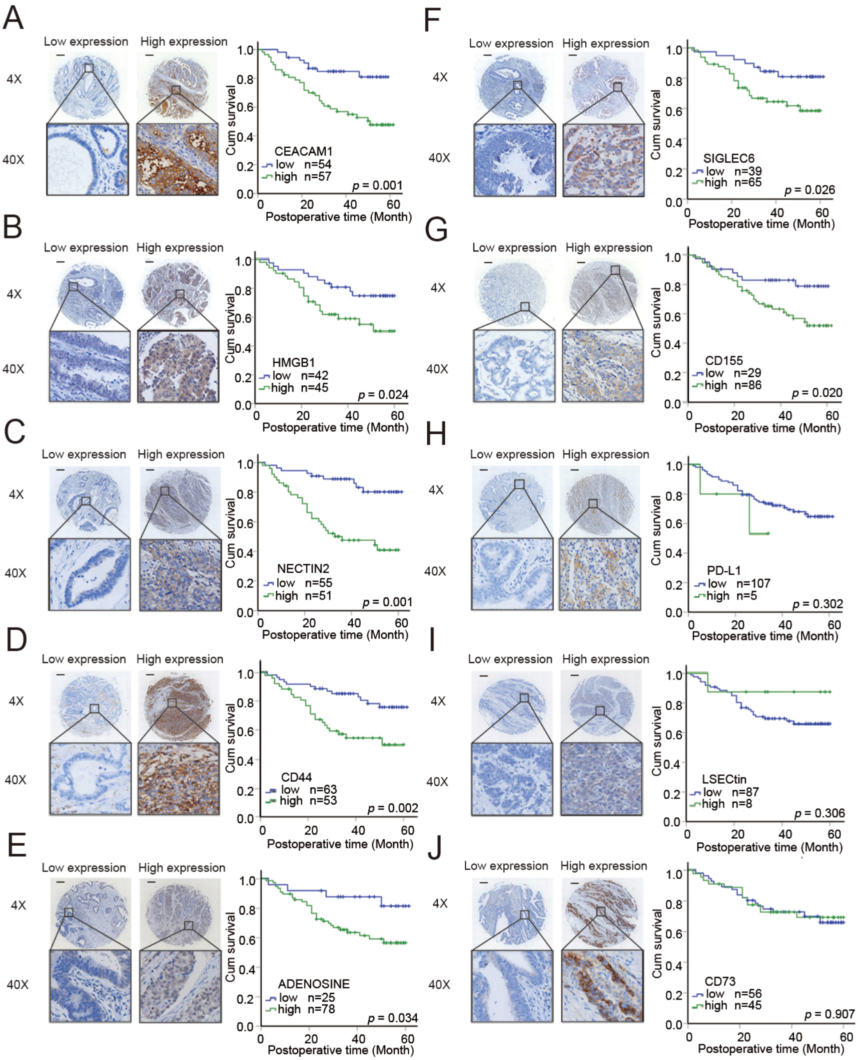

Supplementary Figure 4 TMA results for immune checkpoints (CEACAM1, HMGB1, NECTIN2, CD44, ADENOSINE, SIGLEC6, CD155, PD-L1, LSECTIN and CD73) using IHC, accompanied by their associated overall survival values. Scale bar=200um. *p*-values for all survival analyses have been calculated using the log-rank test.

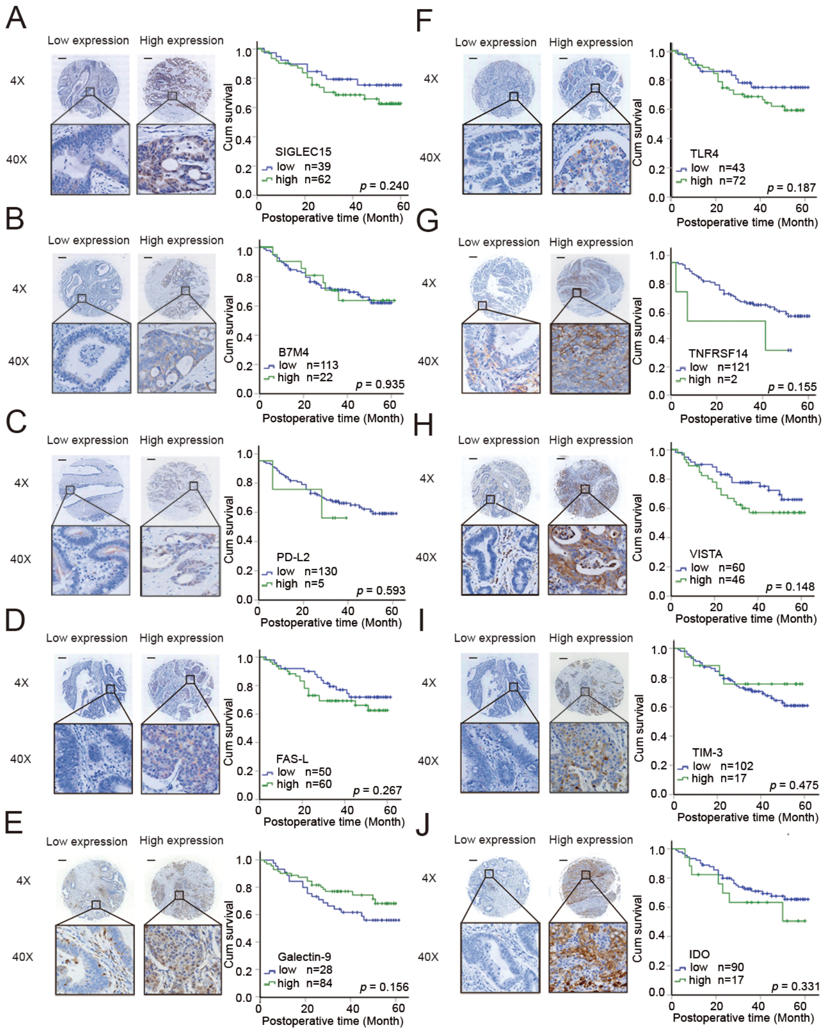

Supplementary Figure 5 TMA results for other immune checkpoints (SIGLEC15, B7M4, PD-L2, FAS-L, GALECTIN9, TLR4, TNFRSF14, VISTA, TIM3 and ID0) using IHC, accompanied by their associated overall survival values. Scale bar=200um. *p*-values for all survival analyses have been calculated using the log-rank test.

Supplementary Figure 6

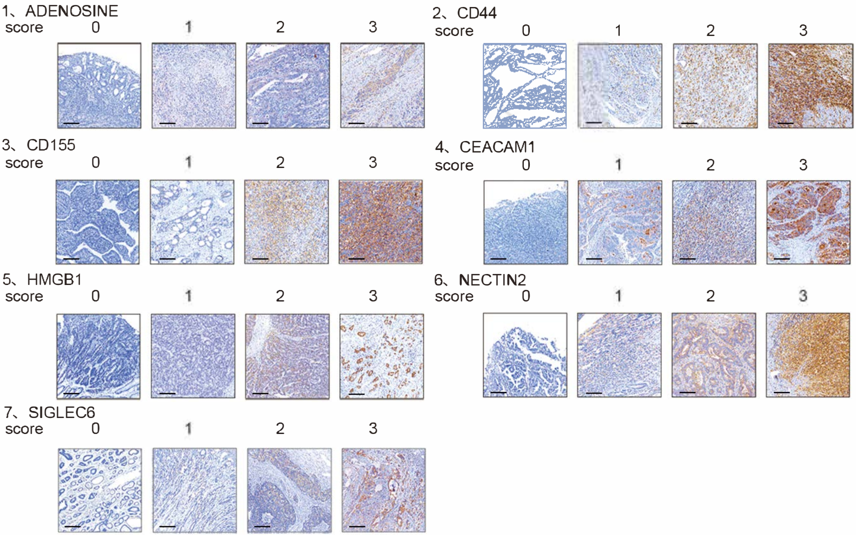

Supplementary Figure 6 Immunohistochemical scoring criteria for the 7 immune checkpoints under x4 magnifi-cation. Scale bar=400um

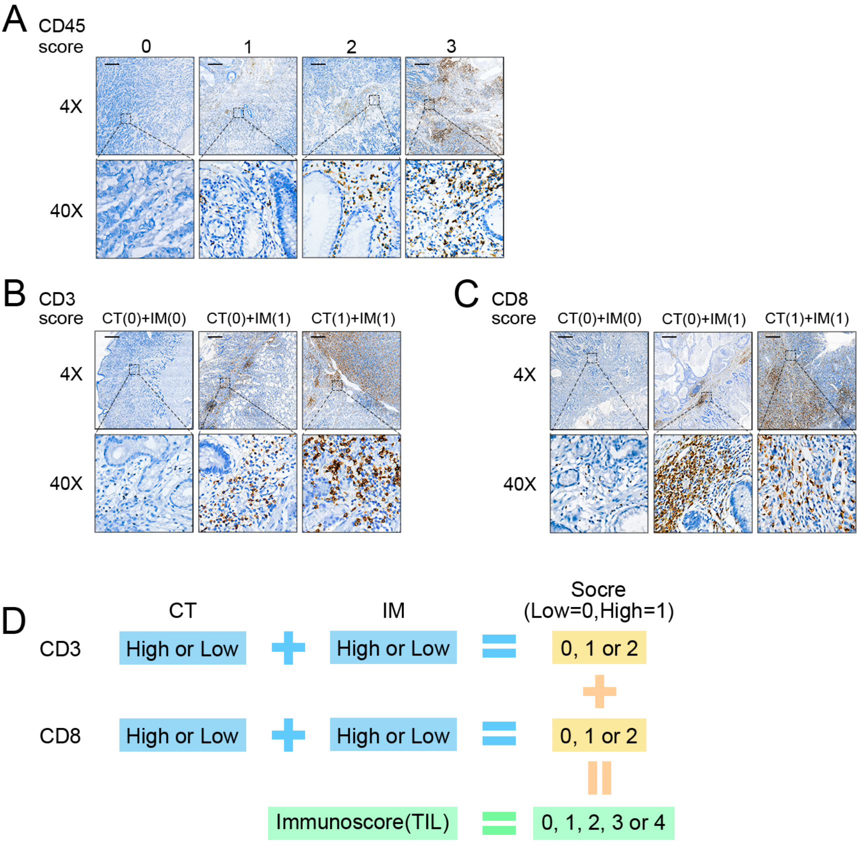

Supplementary Figure 7 (A) Immunohistochemical scoring criteria for CD45. (B-C) Immunohistochemical scoring criteria for CD3CT, CD3IM, CD8CT and CD8IM. (D) Immunoscore definition for TIL. Scale bar=400um.

Supplementary Figure 8

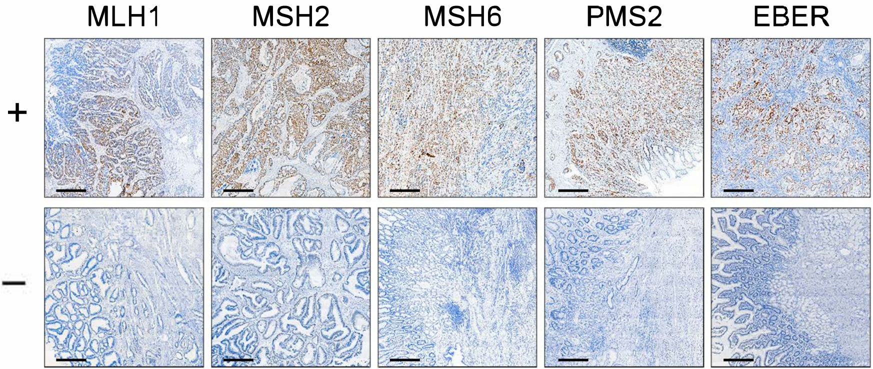

Supplementary Figure 8 Immunohistochemical scoring criteria for MSI and in situ hybridization scoring criteria for EBV status under x4 magnification. Scale bar=400um

Supplementary Figure 9

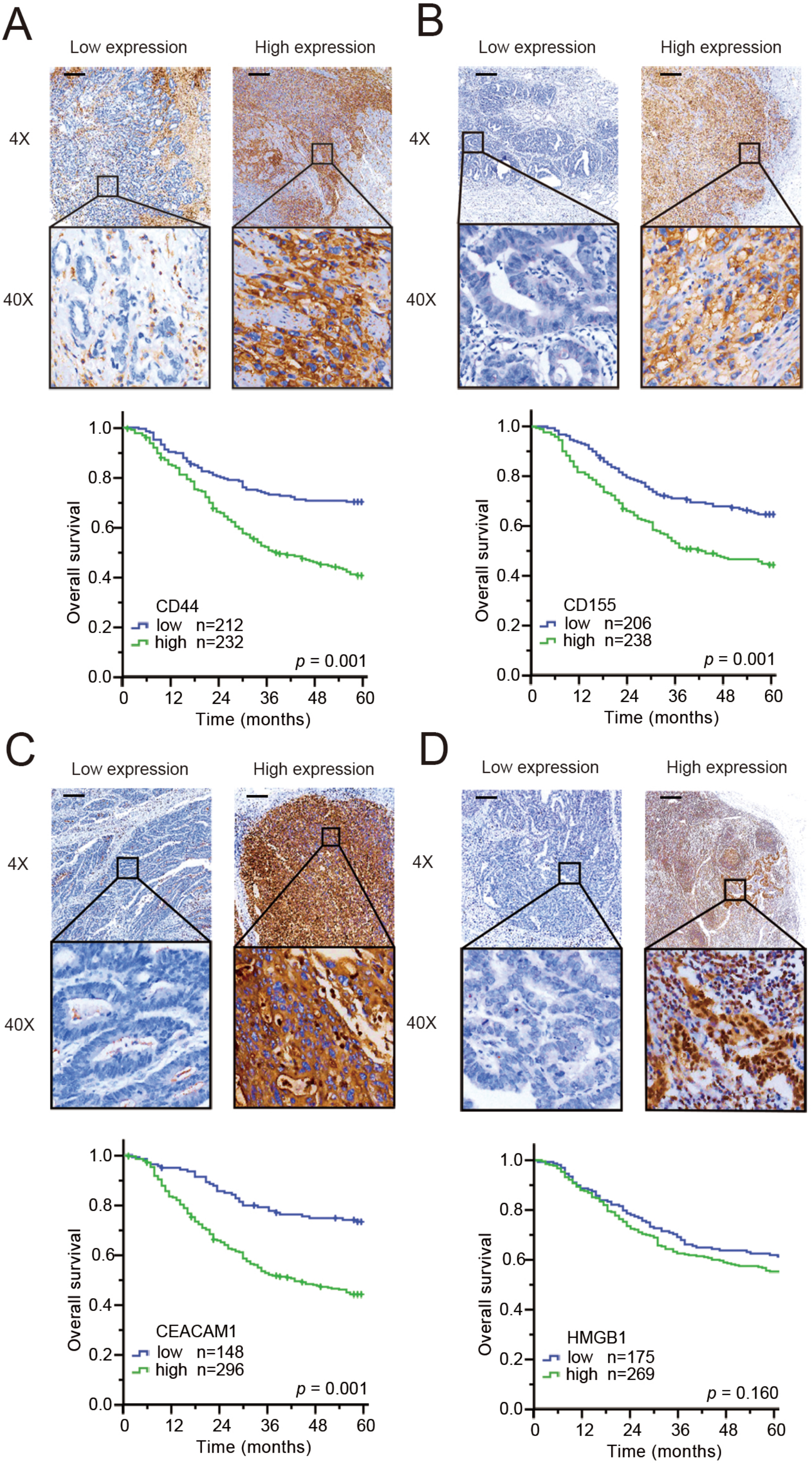

Supplementary Figure 9 IHC results for 4 of 7 immunosuppressive checkpoints (CD44, CD155, CEACAM1 and HMGB1) identified from the 20 immunosuppressive checkpoints, accompanied by their associated overall survival values (n=444). Scale bar=400um. *p*-values for all survival analyses have been calculated using the log-rank test.

Supplementary Figure 10

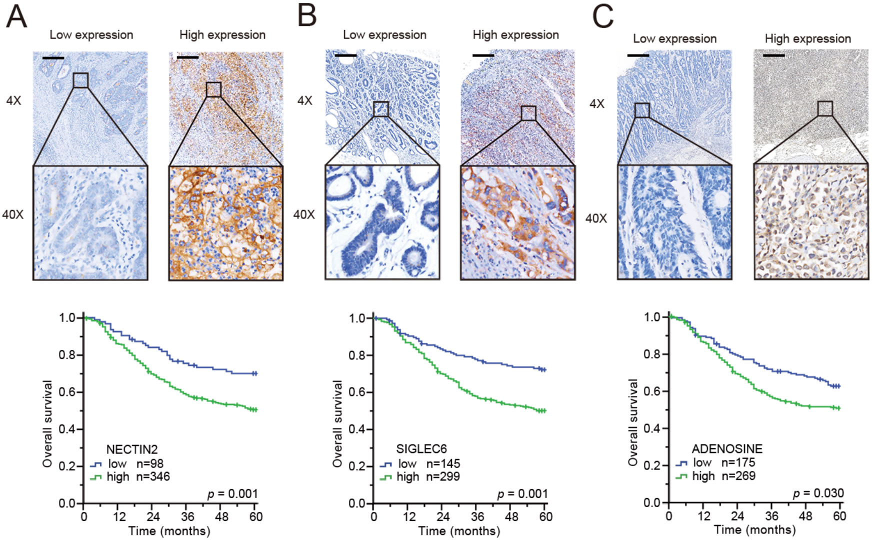

Supplementary Figure 10 IHC results for 3 of 7 immunosuppressive checkpoints (NECTIN2, SIGLEC6 and ADENOSINE) identified from the 20 immunosuppressive checkpoints, accompanied by their associated overall survival values (n=444) Scale bar=400um. *p*-values for all survival analyses have been calculated using the log-rank test.

Supplementary Figure 11

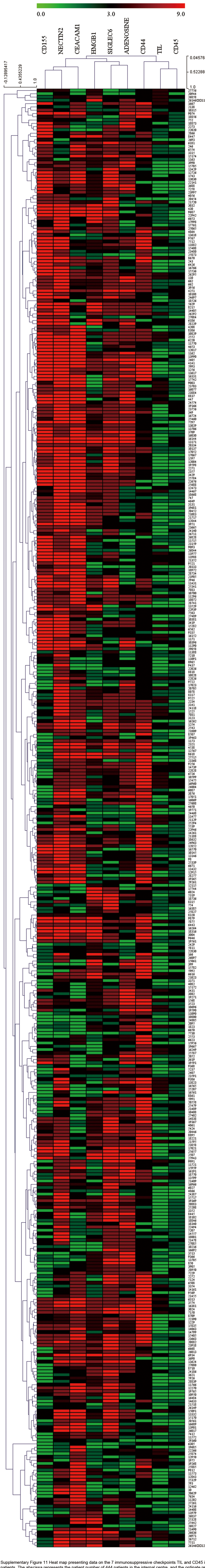

Supplementary Figure 11 Heat map presenting data on the 7 immunosuppressive checkpoints TIL and CD45 in 444 GC patients. The abscissa represents the patient number of 444 patients in the internal centre, and the ordinate represents the expression of CD155, NECTIN2, CEACAM1, HMGB1, SIGLEC6, ADENOSINE, CD44, TIL and CD45.

Supplementary Figure 12

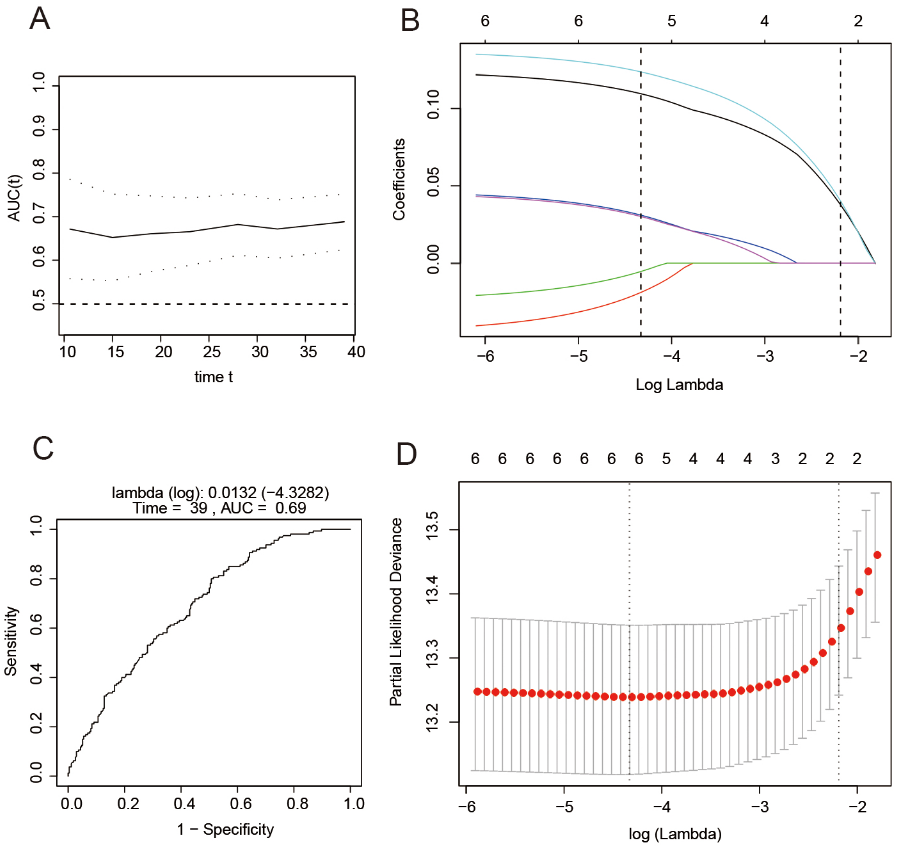

Supplementary Figure 12 Features of the LASSO analysis (AUC, coefficients, sensitivity, and partial likelihood deviance). Error bars indicate estimated 95%CI.

Supplementary Figure 13

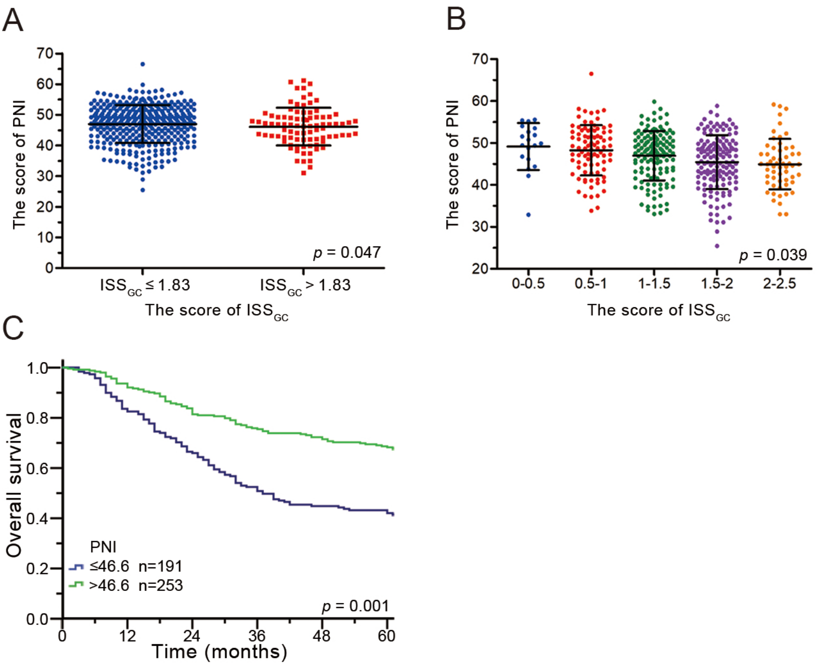

Supplementary Figure 13 Scatter plot of the ISS_{GC} and PNI and the overall survival and PNI. (A) The PNI values of the two groups (ISS_{GC}≤1.83 and ISS_{GC}>1.83) are presented in a scatter plot. Error bars indicate estimated 95%CI (mean ± SEM, N = 444). Statistical significance was determined by a two-tailed unpaired Student's *t*-test. (B) The PNI values of the five groups (0≤ISS_{GC}<0.5, 0.5≤ISS_{GC}<1, 1≤ISS_{GC}<1.5, 1.5≤ISS_{GC}<2 and 2≤ISS_{GC}<2.5) are presented in a scatter plot. Error bars indicate estimated 95%CI (mean ± SEM, N = 444). Statistical significance was determined by a two-tailed unpaired Student's *t*-test. (C) Two groups were created according to the cut-off point for the PNI, and the overall survival of GC patients in these 2 groups was evaluated by Kaplan-Meier curves (N = 444). *p*-values have been calculated using the log-rank test.

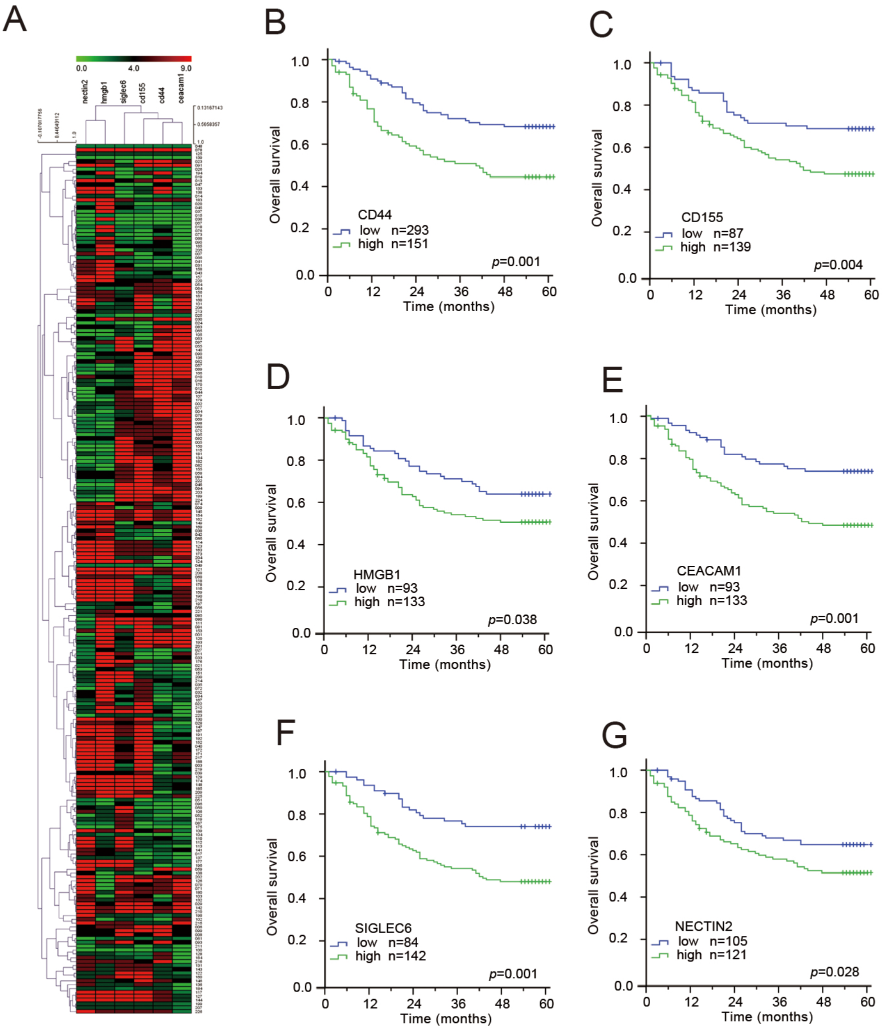

Supplementary Figure 14 External validation. (A) Heat map presenting data for the 6 immunosuppressive checkpoints in 226 GC patients from external centres. (B-G) Overall survival values associated with the 6 immunosuppressive checkpoints in patients from external centres. *p*-values for all survival analyses have been calculated using the log-rank test.

A

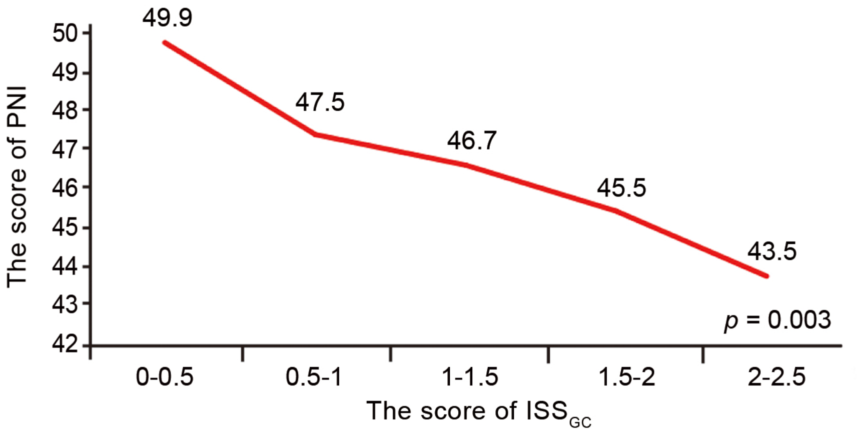

B

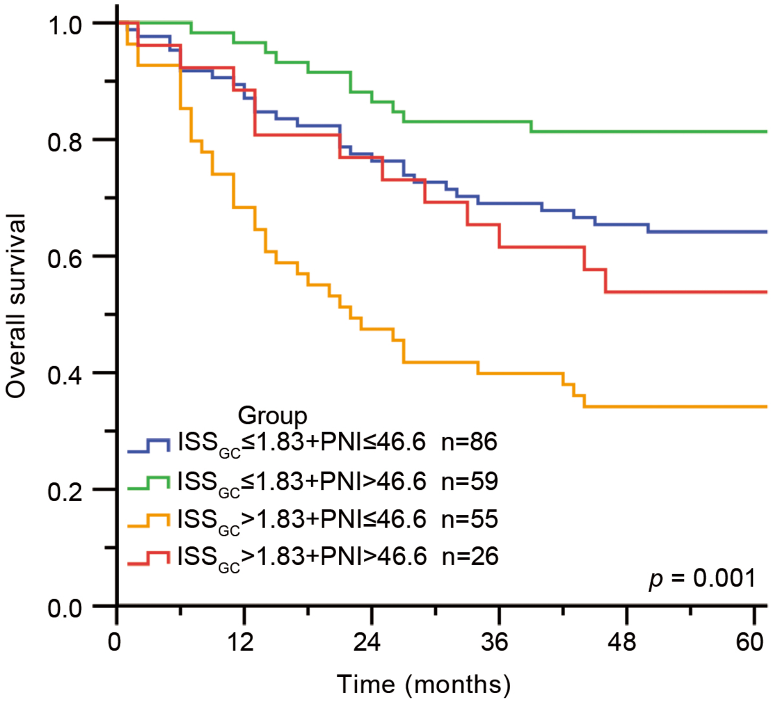

Supplementary Figure 15 External validation. (A) Negative correlation between the PNI and the ISS_{GC} in external cohorts. Statistical significance was determined by a two-tailed paired Student's *t*-test. (B) Four groups were created according to the cut-off points for the ISS_{GC} and the PNI, and the overall survival in GC patients in these 4 groups was evaluated by Kaplan-Meier curves in external cohorts. *p*-values have been calculated using the log-rank test.

Supplementary Figure 16

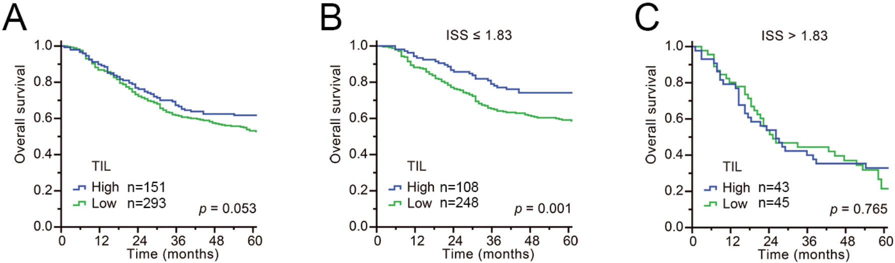

Supplementary Figure 16 Prognostic value of the immune infiltration score according to ISS_{GC} stratification. (A) Overall survival based on the immune infiltration score in 444 GC patients. (B-C) Overall survival based on the immune infiltration score in GC patients after ISS_{GC} stratification. *p*-values for all survival analyses have been calculated using the log-rank test.

Supplementary Table 1. Clinical data of external validation set

Variable	External validation set					
	All patients		Anhui		Qinghai	
**Age(years)**	n=226	%	n=108	%	n=118	%
≤65	161	71.2	67	62.0	94	79.7
>65	65	28.8	41	38.0	24	20.3
**Sex**						
Female	67	29.6	32	29.6	35	29.7
Male	159	70.4	76	70.4	83	70.3
**BMI**						
≤25	103	45.6	0	0.0	103	87.3
>25	15	6.6	0	0.0	15	12.7
Unknown	108	47.8	108	100.0	0	0.0
**Surgery type**						
Open surgery	220	97.3	102	94.4	118	100.0
Laparoscopic surgery	6	2.7	6	5.6	0	0.0
**Resection type**						
Part gastrectomy	161	71.2	66	61.1	95	80.5
Total gastrectomy	65	28.8	42	38.9	23	19.5
**Tumor size**						
≤50mm	156	69.0	77	71.3	79	66.9
>50mm	70	27.4	31	28.7	39	33.1
**Pathological type**						
Adenocarcinoma	181	80.1	84	77.8	97	82.2
Non-adenocarcinoma	5	2.2	1	0.9	4	3.4
Mix	31	13.7	21	19.4	10	8.5
Unknown	9	4.0	2	1.9	7	5.9
**Grade**						
High	34	15.0	7	6.4	27	22.9
Middle	55	24.3	23	21.3	32	27.1
Low	79	35.0	49	45.4	30	25.4
Mix	58	25.7	29	26.9	29	24.6
**Number of lymph node examined**						
≤15	69	30.5	11	10.2	58	49.2
>15	157	69.5	97	89.8	60	50.8
**pT**						
T1	37	16.4	19	17.6	18	15.2
T2	45	19.9	18	16.7	27	22.9
T3	17	7.5	0	0.0	17	14.4
T4	127	56.2	71	65.7	56	47.4
**pN**						
N0	104	46.0	49	45.4	55	46.6
N1	35	15.5	15	13.9	20	17.0
N2	40	17.7	23	21.3	17	14.4
N3	47	20.8	21	19.4	26	22.0
**AJCC7th**						
I	58	25.7	32	29.6	26	22.0
II	66	29.2	23	21.3	43	36.5
III	102	45.1	53	49.1	49	41.5
**Adjuvant Chemotherapy**						
No	113	50.0	60	55.6	53	44.9
Yes	113	50.0	48	44.4	65	55.1
**PNI**						
≤46.6	141	62.4	67	62.0	74	62.7
>46.6	85	37.6	41	38.0	44	37.3
**MSI status**						
MSI-H	65	28.8	47	43.5	18	15.3
MSI-L/MSS	161	71.2	61	56.5	100	84.7
**EBV status**						
negative	217	96.0	101	93.5	116	98.3
positive	9	4.0	7	6.5	2	1.7

Supplementary Table 2. Source of immunosuppressive indicators

Indicators	Literature source
CD73 & ADENOSINE	[1] Xu S , Shao Q Q , Sun J T , et al. Synergy between the ectoenzymes CD39 and CD73 contributes to adenosinergic immunosuppression in human malignant gliomas[J]. Neuro-Oncology, 2013, 15(9):1160-1172.<br>[2] Dahan R , Ravetch J . Co-targeting of Adenosine Signaling Pathways for Immunotherapy: Potentiation by Fc Receptor Engagement[J]. Cancer Cell, 2016, 30(3):369-371.<br>[3] Deaglio S , Dwyer K M , Gao W , et al. Adenosine generation catalyzed by CD39 and CD73 expressed on regulatory T cells mediates immune suppression[J]. Journal of Experimental Medicine, 2007, 204(6):1257-1265.
TIM3 & GLAECTIN-9	[4] Chen Zhu, Kaori Sakuishi, Sheng Xiao, et al. Corrigendum: An IL-27/NFIL3 signalling axis drives Tim-3 and IL-10 expression and T-cell dysfunction[J]. Nature Communications, 2015, 6:6072.<br>[5] Alberto SánchezFueyo, Tian J , Picarella D , et al. Tim-3 inhibits T helper type 1-|mediated auto- and alloimmune responses and promotes immunological tolerance[J]. Nature Immunology, 2003, 4(11):1093-1101.<br>[6] Hongo D , Tang X , Dutt S , et al. Interactions between NKT cells and Tregs are required for tolerance to combined bone marrow and organ transplants[J]. Blood, 2012, 119(6):1581-1589.
HMGB1 & LSECTIN & NECTIN-2	[7] Graham D K , Deryckere D , Davies K D , et al. The TAM family: Phosphatidylserine-sensing receptor tyrosine kinases gone awry in cancer[J]. Nature reviews. Cancer, 2014, 14(12):769-785.<br>[8] Ladoire S , Enot D , Senovilla L , et al. The Presence Of Lc3b Puncta And Hmgb1 Expression In Malignant Cells Correlate With The Immune Infiltrate In Breast Cancer[J]. Autophagy, 2016:00-00.<br>[9] Liu, Di, Lu, Qian, Wang, Xing, et al. LSECtin on tumor-associated macrophages enhances breast cancer stemness via interaction with its receptor BTN3A3[J]. Cell Research, 2019.
FASL & TNF	[10] None. FasL gene knock-down therapy enhances the antiglioma immune response[J]. Neuro-Oncology, 2010, 12(5).<br>[11] Groh V , Smythe K , Dai Z , et al. Fas ligand–mediated paracrine T cell regulation by the receptor NKG2D in tumor immunity[J]. Nature Immunology, 2006, 7(7):755-762.<br>[12] Nakamura M , Nagano H , Sakon M , et al. Role of the Fas/FasL pathway in combination therapy with interferon-α and fluorouracil against hepatocellular carcinoma in vitro[J]. Journal of Hepatology, 2007, 46(1):0-88.
SIGLEC6 & SIGLEC15	[13] Lam K K W , Chiu P C N , Lee C L , et al. Glycodelin-A Protein Interacts with Siglec-6 Protein to Suppress Trophoblast Invasiveness by Down-regulating Extracellular Signal-regulated Kinase (ERK)/c-Jun Signaling Pathway *[J]. Journal of Biological Chemistry, 2011, 286.<br>[14] Angata, T, Margulies, E. H, Green, E. D, et al. Large-scale sequencing of the CD33-related Siglec gene cluster in five mammalian species reveals rapid evolution by multiple mechanisms[J]. Proc Natl Acad Sci USA, 101(36):13251<br>[15] Wang Jun,Sun Jingwei,Liu Linda N et al. Siglec-15 as an immune suppressor and potential target for normalization cancer immunotherapy.[J] . Nat. Med., 2019, 25: 656-666.
TLR4 & IDO & B7M4	[16] Bendickova K , Tidu F , Fric J . Calcineurin-NFAT signalling in myeloid leucocytes: new prospects and pitfalls in immunosuppressive therapy[J]. EMBO Molecular Medicine, 2017: e201707698.<br>[17] Nencioni A , Schwarzenberg K , Brauer K M , et al. Proteasome inhibitor bortezomib modulates TLR4-induced dendritic cell activation.[J]. Blood, 2006, 108(2):551-558.<br>[18] Fric J , Zelante T , Wong A Y , et al. NFAT control of innate immunity[J]. Blood, 2012, 120(7):1380.
CEACAM1	[19] Ding W , Shimada H , Li L , et al. Retinoid agonist Am80-enhanced neutrophil VISTA bactericidal activityarising from granulopoiesis in vitro and in a neutropenic mouse model[J]. Blood, 2013, 121(6):996-1007.<br>[20] Jantscheff, P. Expression of CEACAM6 in Resectable Colorectal Cancer: A Factor of Independent Prognostic Significance[J]. Journal of Clinical Oncology, 2003, 21(19):3638-3646.<br>[21] Brodeur S R , Angelini F , Bacharier L B , et al. C4b-Binding Protein (C4BP) Activates B Cells through the CD40 Receptor[J]. Immunity, 2003, 18(6):837
CD44 & CD155	[22] Paiva B , Corchete L A , Vidriales M B , et al. Phenotypic and genomic analysis of multiple myeloma minimal residual disease tumor cells: A new model to understand chemoresistance[J]. Blood, 2016, 127(15).<br>[23] Co-delivery of paclitaxel and gemcitabine via CD44-targeting nanocarriers as a prodrug with synergistic antitumor activity against human biliary cancer[J]. Biomaterials, 2015, 53:763-774.<br>[24] Gromeier M , Nair S K . Recombinant Poliovirus for Cancer Immunotherapy[J]. Annual Review of Medicine, 2018, 69(1):289-299.
PDL1 & PD-L1	[25] Lim S O , Li C W , Xia W, et al. Deubiquitination and Stabilization of PDL2 by CSN5[J]. Cancer Cell, 2016:S1535610816304974.<br>[26] Robert Lanza, David W. Russell, Andras Nagy, et al. Engineering universal cells that evade immune detection[J]. Nature Reviews Immunology, 2019, (19):723–733.<br>[27] Vari F , Arpon D , Keane C , et al. Immune evasion via PD-1/PD-L1 on NK-cells and monocyte/macrophages is more prominent in Hodgkin lymphoma than DLBCL[J]. Blood, 2018: blood-2017-07-796342.

Supplementary Table 3. Spearman correlation analysis of training set (n=444)

Correlation coefficient	SIGLEC6	CD44	CD155	HMGB1	NECTIN2	CEACAM1	ADENOSINE
SIGLEC6	1	0.061	0.192**	0.297**	0.202**	0.160**	0.328**
CD44	0.061	1	0.052	0.094*	0.045	-0.033	0.133**
CD155	0.192**	0.052	1	0.205**	0.464**	0.342**	0.135**
HMGB1	0.297**	0.094*	0.205**	1	0.158*	0.087	0.179**
NECTIN2	0.202**	0.045	0.464**	0.158*	1	0.384**	0.105*
CEACAM1	0.160**	-0.033	0.342**	0.087	0.384**	1	0.008
ADENOSINE	0.328**	0.133**	0.135**	0.179**	0.105*	0.008	1

**p<0.01 *p<0.05

Supplementary Table 4. Spearman correlation analysis of training set(n=444)

Variable	TIL		CD45	
	r	P	r	P
SIGLEC6	-0.008	0.867	0.004	0.938
CD44	0.073	0.122	0.060	0.208
CD155	0.061	0.200	0.046	0.336
HMGB1	-0.205	0.593	0.057	0.232
NECTIN2	0.095	0.057	0.086	0.071
CEACAM1	0.059	0.214	0.000	0.993

Supplementary Table 5. Cox regression analysis of training set(n=444)

Variable	Univariate Model				Reduced Multivariate Model			
	HR	95%CI		P	OR	95%CI		P
**Age(years)**								
≤65	Ref							
>65	1.33	1.01	1.75	0.043				
**Sex**								
Female	Ref							
Male	1.02	0.74	1.40	0.911				
**BMI**								
≤25	Ref							
>25	0.79	0.52	1.19	0.261				
**Surgery type**								
Open surgery	Ref							
Laparoscopic surgery	0.74	0.33	1.67	0.466				
**Resection type**				0.209				
Part gastrectomy	Ref							
Total gastrectomy	1.23	0.93	1.63	0.144				
**Tumor size**								
≤50mm	Ref				Ref			
>50mm	1.87	1.41	2.48	<0.001	1.65	1.23	2.23	0.001
**Pathological type**				0.810				
Adenocarcinoma	Ref							
Non-adenocarcinoma	0.85	0.44	1.66	0.637				
Mix	0.90	0.59	1.37	0.630				
**Number of Lymph node examined**								
≤15	Ref							
>15	0.99	0.51	1.93	0.980				
**AJCC7th**				<0.001				<0.001
I	Ref				Ref			
II	2.10	1.08	4.07	0.028	2.37	1.21	4.64	0.012
III	5.18	2.81	9.57	<0.001	5.14	2.75	9.60	<0.001
**MSI status**								
MSI-H	Ref							
MSI-L/MSS	0.99	0.73	1.36	0.965				
**EBV status**								
negative	Ref							
positive	0.60	0.27	1.35	0.215				
**Adjuvant Chemotherapy**								
No	Ref				Ref			
Yes	0.61	0.46	0.81	0.001	0.51	0.38	0.69	<0.001
**PNI**								
≤46.6	Ref							
>46.6	0.49	0.37	0.64	<0.001	0.64	0.48	0.85	0.002
**ISS_{GC}**								
≤1.83	Ref							
>1.83	2.66	1.97	3.58	<0.001	2.10	1.55	2.84	<0.001

Supplementary Table 6. Correlation analysis between Signature and PNI

Variable	0<score≤0.5	0.5<score≤1	1<score≤1.5	1.5<score≤2	2<score≤2.5	P†	P††
PNI	49.17±5.614	48.27±6.019	46.97±5.907	45.43±6.402	44.94±6.022	0.001	0.005

P† Spearman's test between lasso score(Categorical variables) and blood marker(Continuous variable)

P†† Kruskal-Wallis test

References

1 Xu S, Shao Q Q, Sun J T, et al. Synergy between the ectoenzymes CD39 and CD73 contributes to adenosinergic immunosuppression in human malignant gliomas. Neuro-Oncology **15(9)**:1160-1172 (2013).

2 Dahan R, Ravetch J. Co-targeting of Adenosine Signaling Pathways for Immunotherapy: Potentiation by Fc Receptor Engagement. Cancer Cell **30(3)**:369-371 (2016).

3 Deaglio S , Dwyer K M , Gao W , et al. Adenosine generation catalyzed by CD39 and CD73 expressed on regulatory T cells mediates immune suppression. Journal of Experimental Medicine, **204(6)**:1257-1265 (2007).

4 Chen Zhu, Kaori Sakuishi, Sheng Xiao, et al. Corrigendum: An IL-27/NFIL3 signalling axis drives Tim-3 and IL-10 expression and T-cell dysfunction. Nature Communications **6**:6072 (2015).

5 Alberto SánchezFueyo, Tian J, Picarella D, et al. Tim-3 inhibits T helper type 1-|mediated auto- and alloimmune responses and promotes immunological tolerance. Nature Immunology **4(11)**:1093-1101 (2003).

6 Hongo D, Tang X, Dutt S, et al. Interactions between NKT cells and Tregs are required for tolerance to combined bone marrow and organ transplants. Blood **119(6)**:1581-1589 (2012).

7 Graham D K, Deryckere D, Davies K D, et al. The TAM family: Phosphatidylserine-sensing receptor tyrosine kinases gone awry in cancer. Nature reviews. Cancer **14(12)**:769-785 (2014).

8 Ladoire Sylvain, Enot David, Senovilla Laura et al. The presence of LC3B puncta and HMGB1 expression in malignant cells correlate with the immune infiltrate in breast cancer. Autophagy **12**: 864-75 (2016).

9 Liu Di, Lu Qian, Wang Xing et al. LSECtin on tumor-associated macrophages enhances breast cancer stemness via interaction with its receptor BTN3A3.Cell Res. **29**: 365-378 (2019).

10 None. FasL gene knock-down therapy enhances the antiglioma immune response. Neuro-Oncology **12**: 482-9 (2010).

11 Groh V, Smythe K, Dai Z, et al. Fas ligand–mediated paracrine T cell regulation by the receptor NKG2D in tumor immunity. Nature Immunology **7(7)**:755-762 (2006).

12 Nakamura M, Nagano H, Sakon M, et al. Role of the Fas/FasL pathway in combination therapy with interferon-α and fluorouracil against hepatocellular carcinoma in vitro. Journal of Hepatology, 2007, 46(1):0-88.

13 Lam Kevin K W, Chiu Philip C N, Lee Cheuk-Lun et al. Glycodelin-A protein interacts with Siglec-6 protein to suppress trophoblast invasiveness by down-regulating extracellular signal-regulated kinase (ERK)/c-Jun signaling pathway.bJ. Biol. Chem. **286**: 37118-27 (2011).

14 Angata Takashi, Margulies Elliott H, Green Eric D et al. Large-scale sequencing of the CD33-related Siglec gene cluster in five mammalian species reveals rapid evolution by multiple mechanisms. Proc. Natl. Acad. Sci. U.S.A. **101**: 13251-6 (2004).

15 Wang Jun, Sun Jingwei, Liu Linda N et al. Siglec-15 as an immune suppressor and potential target for normalization cancer immunotherapy. Nat. Med. **25**: 656-666 (2019).
16 Bendickova Kamila, Tidu Federico, Fric Jan, Calcineurin-NFAT signalling in myeloid leucocytes: new prospects and pitfalls in immunosuppressive therapy. EMBO Mol Med **9**: 990-999 (2017).
17 Nencioni A, Schwarzenberg K, Brauer K M, et al. Proteasome inhibitor bortezomib modulates TLR4-induced dendritic cell activation. Blood **108(2)**:551-558 (2006).
18 Fric J, Zelante T, Wong A Y, et al. NFAT control of innate immunity. Blood **120(7)**:1380 (2012).
19 Ding W, Shimada H, Li L, et al. Retinoid agonist Am80-enhanced neutrophil VISTA bactericidal activityarising from granulopoiesis in vitro and in a neutropenic mouse model. Blood **121(6)**:996-1007 (2013).
20 Jantscheff, P. Expression of CEACAM6 in Resectable Colorectal Cancer: A Factor of Independent Prognostic Significance. Journal of Clinical Oncology **21(19)**:3638-3646 (2003).
21 Brodeur S R, Angelini F, Bacharier L B, et al. C4b-Binding Protein (C4BP) Activates B Cells through the CD40 Receptor. Immunity **18(6)**:837 (2003).
22 Paiva B, Corchete L A, Vidriales M B, et al. Phenotypic and genomic analysis of multiple myeloma minimal residual disease tumor cells: A new model to understand chemoresistance. Blood **127(15)** (2016).
23 Co-delivery of paclitaxel and gemcitabine via CD44-targeting nanocarriers as a prodrug with synergistic antitumor activity against human biliary cancer. Biomaterials **53**:763-774 (2015).
24 Gromeier M, Nair S K. Recombinant Poliovirus for Cancer Immunotherapy. Annual Review of Medicine **69(1)**:289-299 (2018).
25 Lim Seung-Oe, Li Chia-Wei, Xia Weiya, et al. Deubiquitination and Stabilization of PD-L1 by CSN5. Cancer Cell **30**: 925-939 (2016).
26 Robert Lanza, David W. Russell, Andras Nagy, et al. Engineering universal cells that evade immune detection. Nature Reviews Immunology **19**:723–733 (2019).
27 Vari Frank, Arpon David, Keane Colm, et al. Immune evasion via PD-1/PD-L1 on NK cells and monocyte/macrophages is more prominent in Hodgkin lymphoma than DLBCL. Blood **131**: 1809-1819 (2018).
