# Supplementary material for: An immune checkpoint score system for prognostic evaluation and adjuvant chemotherapy selection in gastric cancer
Source: Nat Commun. 2020 Dec 11;11:6352. doi: 10.1038/s41467-020-20260-7 (PMC7732987; doi:10.1038/s41467-020-20260-7)
Supplement: Supplementary file 3 — Descriptions of Additional Supplementary Files [file 41467_2020_20260_MOESM3_ESM.pdf]

## Descriptions of Additional Supplementary Files

**Supplementary data 1**

Description: The survival and clinicopathological data and the Immunohistochemical score of 124 GC patients in tissue microarray.

**Supplementary data 2**

**Description:** The survival and clinicopathological data and the Immunohistochemical score of 444 GC patients in the internal center.

**Supplementary data 3**

**Description:** The survival and clinicopathological data and the Immunohistochemical score of 226 GC patients in the external center.
